# Supplementary material for: Conditional chemoconnectomics (cCCTomics) as a strategy for efficient and conditional targeting of chemical transmission
Source: eLife. 2024 Apr 30;12:RP91927. doi: 10.7554/eLife.91927 (PMC11060718; doi:10.7554/eLife.91927)
Supplement: Figure 3—source data 3. [file elife-91927-fig3-data3.zip › Figure 3 source data3 part 1.zip!/Figure 3-Source data 3-part1/1071f2-1-CRISPResso_Report_ztwktvfp.zip!/CRISPResso_on_1071f2_4/2a.Nucleotide_percentage_quilt.pdf]

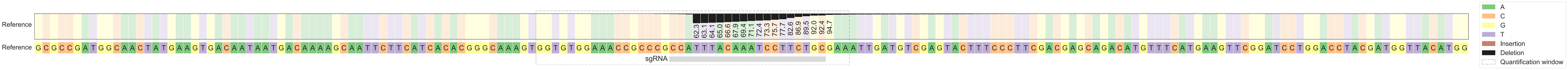
Reference
62.3 63.1 64.1 65.0 66.6 67.9 69.4 71.1 72.4 73.3 75.7 77.7 82.6 86.9 89.5 92.0 92.4 94.7
Reference
GCGCCGATGGCAACTATGAAGTGACAATAATGACAAAAGCAATTCTTCATCACACGGGCAAAGTGGTGTGGAAACCGCCCGCCATTTACAAATCCTTCTGCGAAATTGATGTCGAGTACTTTCCCTTCGACGAGCAGACATGTTTCATGAAGTTCGGATCCTGGACCTACGATGGTTACATGG
sgRNA
A
C
G
T
Insertion
Deletion
Quantification window
